# Small rodents as paratenic or intermediate hosts of carnivore parasites in Berlin, Germany

**DOI:** 10.1371/journal.pone.0172829

**Published:** 2017-03-09

**Authors:** Jürgen Krücken, Julia Blümke, Denny Maaz, Janina Demeler, Sabrina Ramünke, Daniela Antolová, Roland Schaper, Georg von Samson-Himmelstjerna

**Affiliations:** 1 Institute for Parasitology and Tropical Veterinary Medicine, Freie Universität Berlin, Berlin, Germany; 2 Institute of Parasitology, Slovak Academy of Sciences, Košice, Slovak Republic; 3 Bayer Animal Health GmbH, Leverkusen, Germany; Tulane University, UNITED STATES

## Abstract

Rodents are important intermediate and paratenic hosts for carnivore parasites, including the important zoonotic agents *Toxoplasma*, *Echinococcus* and *Toxocara*. Monitoring of such parasites in rodents can be used to detect increasing risks for human and veterinary public health. Rodents were trapped at four sites in Berlin, two near the city center, two at the periphery. PCRs were conducted to detect Coccidia (target ITS-1) and specifically *Toxoplasma gondii* (repetitive element) in brain and ascarids (ITS-2) in muscle or brain tissue. During necropsies, metacestodes were collected and identified using ITS-2 and 12S rRNA PCRs. An ELISA to detect antibodies against *Toxocara canis* ES antigens was performed. Within the 257 examined rodents, the most frequently observed parasite was *Frenkelia glareoli* predominantly found in *Myodes glareolus*. *T*. *gondii* was only detected in 12 rodents and *Microtus* spp. (although strongly underrepresented) had a significantly increased chance of being positive. Neither *Echinococcus* nor typical *Taenia* parasites of dogs and cats were found but *Mesocestoides litteratus* and *Taenia martis* metacestodes were identified which can cause severe peritoneal or ocular cysticercosis in dogs, primates and humans. Using PCR, the ascarids *T*. *canis* (n = 8), *Toxocara cati* (4) and *Parascaris* sp. (1) were detected predominantly in muscles. Seroprevalence of *T*. *canis* was 14.2% and ELISA was thus more sensitive than PCR to detect infection with this parasite. Non-parametric multidimensional scaling and cluster analysis revealed that parasite communities could be grouped into an urban and a peri-urban cluster with high frequency of ascarid-positive rodents in urban and high frequency of *F*. *glareoli* in peri-urban sites. Prevalence rates of parasites in rodents with potential impact for human or veterinary public health are considerable and the monitoring of transmission cycles of carnivore parasites in intermediate rodent hosts is recommended to estimate the health risks arising from wild and domesticated carnivores.

## Introduction

In many urban environments, the density of carnivores has dramatically increased in the last decades. In central Europe, this is not only due to increased numbers of domesticated cats and dogs, but also a result of establishment of urban and peri-urban populations of red foxes (*Vulpes vulpes*) and neozoic racoons (*Procyon lotor*) which often considerably exceed that in rural or natural habitats [1]. In addition, racoon dogs (*Nyctereutes procyonoides*) are another neozoan species that has rapidly expanded its geographical range and shares many parasites it with other carnivores [2]. Populations of many species of birds of prey such as common buzzards (*Buteo buteo*) and falcons (*Falco peregrinus* and *Falco tinnunculus*) have also at least partially recovered and several species no longer avoid the vicinity of humans or villages. This allows increased predator-prey interaction and increases the transmission of pathogens between predators and their prey, which are predominantly small rodents and birds. High population densities of carnivores, in particular of foxes and racoons, surely pose increased health risks not only for companion animals but also for humans since important parasites of carnivores such as *Toxoplasma gondii*, *Toxocara* spp. and *Echinococcus multilocularis* are also highly relevant zoonoses [3].

Small rodents are reservoirs for many bacterial and viral pathogens that affect humans and companion animals [4]. Among the most important to mention are presumably several species of *Rickettsia*, *Borrelia*, *Leptospira* and *Bartonella*. Rodents are also considered to be important paratenic and intermediate hosts of protozoan and helminth parasites affecting cats, dogs and wild carnivores as recently elaborated by Duscher *et al*. [3] (see in particular Fig 1 in Duscher et al.). Prevalences of parasites with obligate intermediate hosts can be considered to be particularly high in ecosystems with intact and close predator/prey relationships. However, stray as well as pet dogs and cats do often feed on wild rodents and this bears the risk that transmission of such parasites spills over from a sylvatic into a domestic or even synanthropic cycle and that incidences in humans increase [3]. For parasites with facultative intermediate or paratenic hosts, domestic/synanthropic cycles can establish more easily but rodent populations can also amplify transmission of these pathogens. In addition to rodent density, other ecological factors such as species richness and species composition can have strong effects on rodent impact on human and animal health [5]. This is further complicated by the fact that rodent communities are strongly influenced by human activities and that with increasing urbanization the species richness decreases and a few generalist species dominate [5]. Since rodent species differ in their permissibility for different parasites, such changes in host diversity can of course be expected to shift parasite diversity and abundance as well.

**Fig 1:**
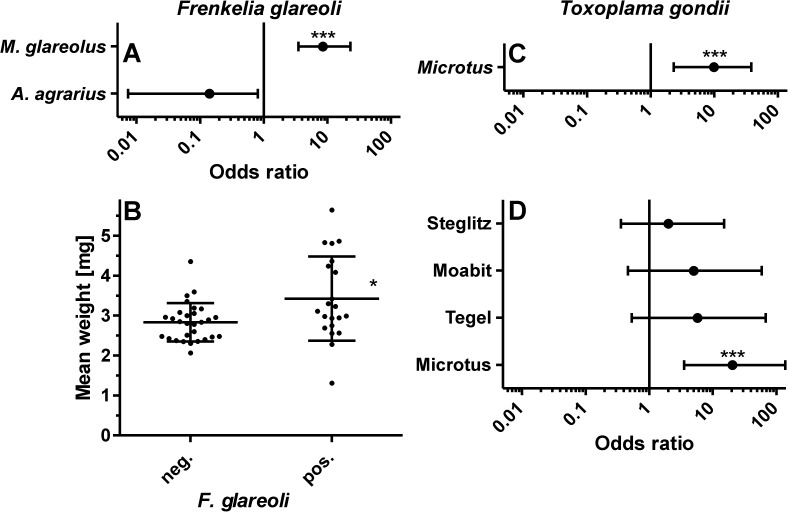
Statistical analysis of parameters affecting the probability to detect DNA from Coccidia. (A) Odds ratios with 95% confidence intervals are shown for variables determining the risk for detection of *Frenkelia glareoli* in *Myodes glareolus* and *Apodemus agrarius* compared to *Apodemus flavicollis*. A model considering only the rodent species as explanatory variables is presented. Odds ratios for *Apodemus sylvaticus*, *Microtus agrestis* and *Microtus arvalis* were 9.3×10^−8^ and were omitted from the figure due to the fact that the parasite was not found in any of these animals and the resulting very wide confidence intervals. (B) Distribution of dry eye lens weights, as an indirect indicator for age, in *M*. *glareolus* with and without *F*. *glareoli* infection. Only animals trapped in 2011 were considered since eye lens weights were not determined in 2010. Individual points representing means of two eye lenses are shown together with means ± SD for all animals in the group. *, p < 0.05 in a Mann-Whitney U test. (C, D). Odds ratios with 95% confidence intervals for the probability to detect *Toxoplasma gondii* DNA in the brain by PCR are illustrated. The model in (C) considered only the rodent genus, the model in (D) genus and study location. *Myodes* is not shown since *T*. *gondii* was not found in these rodents. The odds ratios for *Myodes* spp. was 7.0×10^−8^ and the 95% confidence interval was very wide. Reference categories were *Apodemus* for genus and Gatow for study location. All odds ratios and significance levels were calculated by logistic regression followed by t test to determine the significance of the effects. ***, p < 0.001.

Important parasites transmitted between small rodents and carnivores include three main groups of parasites: (i) the Coccidia such as *T*. *gondii*, *Neospora caninum* and several species of the genera *Cystoisospora*, *Hammondia* and *Sarcocystis*; (ii) the Cestoda comprising species of the genera *Echinococcus*, *Taenia* and *Mesocestoides*; (iii) the Ascarida predominantly represented by *Toxocara canis* and *Toxocara cati*. From a clinical point of view, most of these pathogens cause only benign to moderate signs of pathology in cats and dogs with *N*. *caninum* and *Toxocara* spp. as the most pathogenic infections for the definitive hosts. Many of the infections are predominantly found in young puppies and kittens, but particularly the cestode species and *Sarcocystis* spp. are frequently also found in adult pets. However, in recent years more attention has also been attributed to *Toxocara* infections in older animals, which have apparently been underestimated for a long time [6]. Since in particular *T*. *gondii*, *Echinococcus* spp. and *Toxocara* spp. are important zoonotic agents, the role of small rodents in the epidemiology of these pathogens is also of importance for human medicine in a modern One-Health concept. Echinococcosis is listed by the WHO among the neglected tropical diseases and Toxocarosis as well as Toxoplasmosis are considered to be important neglected diseases of poverty in both, the USA and Europe [7–9]. Among the most dramatic consequences these parasitoses may have on humans are severe neurological disorders including (meningo-) encephalitis, epilepsy, stroke and dementia [10–12] as well as ocular complications [13, 14]. Toxocarosis and Toxoplasmosis have also been reported to significantly change the behavioral pattern of rodents shifting the balance of risk/fear behavior [15–18]. They also lead to decreased performance in several cognitive tests in humans [18–22]. Apparently, the impact of the chronic form of these diseases on human health has been underestimated in the past.

Overall, our current knowledge regarding prevalence of these pathogens in small rodents is relatively sparse. Particularly in urban and peri-urban biotopes, where numbers of domestic dogs and cats can be quite high, relevant data are still scarce. It was therefore aimed to compare urban and peri-urban sites in Berlin for the presence of parasites known to be transmitted between carnivores and rodents. The study focused on parasites with importance for human, feline or canine health and included coccidian, ascarid and cestode parasites.

## Materials and methods

### Ethics statement

All animal experiments were in accordance with the German Animal Protection Act (“Tierschutzgesetz”) and the European Union directive 2010/63/EU and were approved by the Landesamt für Gesundheit und Soziales (LAGeSo) Berlin under the registration number G 0256/10. A permission to trap and euthanize protected rodents (*Apodemus sylvaticus*, *Apodemus flavicollis* and *Apodemus agrarius*) was granted by the Senatsverwaltung für Stadtentwicklung Berlin according to §45 no. 3 “Bundesnaturschutzgesetz” under the reference number I E 210(V)–OA-SG / LSG2a/602; OA-AS/G/825.

### Capture of wild rodents

Rodents were caught in live traps at four different sites in Berlin in November 2010 and from April to November 2011. Two of the sites (forests in Tegel, GPS coordinates 52.605857, 13.271460, and on a military area in Gatow, GPS coordinates 52.469455, 13.140998) were located in the periphery of the city (peri-urban) whereas the other two sites (park in Steglitz, GPS coordinates 52.453881, 13.302522, and garden/backyard in Moabit, GPS coordinates 52.521426, 13.359520) were directly in the city (urban). At every site, traps were placed every six weeks for three consecutive nights (Monday to Thursday). Sites were visited in blocks with one site per week, i.e. starting in 2010 in Tegel, followed by Moabit and Steglitz. In 2011 the first sampling was performed in Gatow, followed by Tegel, Moabit and Steglitz. Between the four weeks of trapping in each block, trapping was interrupted for 2 weeks. In total, each site was visited 6 times in 2011 with the exception of Gatow which was visited 7 times. At every site, 30 Longworth Traps (NHBS, Devon, UK) and 10 traps for rats filled with cotton pads, rodent pellets and pieces of apples were distributed over the area. In July 2011, the number of Longworth Traps was increased to 50 in Gatow, Tegel and 40 in Steglitz to increase the number of trapped rodents. In Moabit the space was too limited to increase the number of traps. Traps were activated in the evening and controlled in the morning but were inactive during daytime. If rodents were found in the traps in the morning, they were anaesthetized using 0.1 mg/g Ketamin and 0.012 mg/g Xylazin intraperitoneal (i.p.) followed by cervical dislocation and cardiac bleeding into serum tubes (Sarstedt). Animals were kept on heat packs to preserve body temperature (to improve recovery of parasites) and transferred to the Institute for Parasitology and Tropical Veterinary Medicine for necropsies. Some animals (n = 16, all *A*. *agrarius* in Steglitz) had to be released after capturing because it was not possible to perform more than eight necropsies per day.

### Study sites

The military area in Gatow was characterized by a small wood area comprised of broad-leafed trees and conifers. Adjacent to the woods, traps were also placed in grassland with high vegetation. The area was only rarely entered by humans and vegetation was barely disturbed by human influences in the last years.

The study site in the forest of Tegel was close to the forestry office. Traps were placed on the area around the office building and in the adjacent forest. They were placed in deciduous forest and shrubs on both sides of a small road (approximately 100 m) predominantly used by bicyclers and pedestrians.

The site in Steglitz was located within a large park area in a residential area. The park can only be entered through two entrances and is surrounded by a fence. There is no access for dogs to this area (prohibited and controlled at the entrance gates). The particular study site in the park consisted of an area with broad-leafed trees and dense underbrush adjacent to a lawn. The majority of the ground was covered with ivy. The whole area was watered frequently. In the vicinity, there were a few greenhouses that were used for cultivating a variety of plants for subsequent planting in the park.

The trapping site in Moabit was within a very urbanized area immediately between several apartment blocks. There were also additional gardens and backyards adjacent to the study site that could not be used to place traps. Within the study site, there was a lawn, which was surrounded by flowerbeds of different vertical levels and ivy. A compost heap, housing some rats, was removed some weeks before the study period.

### Necropsies

After collecting all visible ectoparasites under a stereo microscope (for another study), a complete necropsy was conducted. Rodents were sexed and depending on the juvenile vs. adult fur, size and habitus as well as sign of sexual activity classified as juveniles, subadults or adult, with subadults as full-grown individuals without the following signs of sexual activity. Males with descended testicles [23] or females with embryos or scars in the uterus or showing lactation were always considered adult. With regard to the study presented here, one complete hind leg and the brain were removed and immediately frozen at -80°C. In a few cases, metacestodes (tetrathyridia, taeniid or other cestode larvae) were identified in the body cavity, removed and stored in 80% ethanol until isolation of DNA. Livers and lungs were inspected for any signs of potential infections with *E*. *multilocularis*. Suspicious tissue sites were extracted and stored in ethanol (80%).

In uncertain cases, especially for *Microtus* spp., species determination of rodents was confirmed according to teeth morphology [24]. For the estimation of rodent age, the mean dry weight of both eye lenses was used. According to the recommended procedure in Morris [25], eyes were removed and fixed in 10% formalin for one week. Subsequently, lenses were removed, cleaned and dried at 80°C for 48 h. Then, both lenses were immediately weighted using an analytical balance (Discovery DV215CDM, Ohaus with a readability of 0.01 mg and a repeatability of 0.02 mg). Morphologic species determination of larval cestodes was performed under a microscope with up to 1000× magnification following the differentiation guides according to Ryzhikov *et al*. [26] and Schmidt [27].

### Serological analyses

Blood samples of captured rodents in serum tubes were centrifuged at 10000×g for 5 min to obtain serum supernatants. Serum samples were analyzed by ELISA regarding presence of antibodies against *T*. *canis* as described previously [28] The ELISA for the detection of antibodies against *T*. *canis* uses excretory-secretory (ES) antigen and showed no cross reactivity with other ascarid species [29]. Parasite free laboratory mice and laboratory mice experimentally infected with *T*. *canis* were used to provide negative and positive control sera, respectively.

### DNA extraction

DNA was extracted from 30 mg of the *musculus rectus femoris*, a cross section of the brain, pieces of liver tissue suspected of being affected by alveolar echinococcosis or free metacestodes using the Maxwell^®^ 16 Research System (Promega) and the Maxwell^®^ 16 Lev Blood DNA kit (Promega). Purification was conducted using the Maxwell^®^ program “BLOOD DNA”. DNA was eluted in 50 μl elution buffer included in the kit.

### PCR detection of pathogens

PCRs were adapted using published primer sequences [30–36]. All PCRs for detection of pathogens were performed by running a no-template (negative) and a positive control, containing a defined number of plasmid molecules (10–40 depending on the PCR) with the target sequence as insert in parallel. PCR reactions contained 0.2 mM dNTPs, 0.3 μM of each primer, 0.4 U Phusion Hot Start II High-Fidelity DNA polymerase (Thermo Scientific) and 100 ng template DNA in 20 μl 1×HF buffer. After denaturation at 98°C for 30 s, a target-specific number of PCR cycles was conducted. Cycles consisted of denaturation at 98°C for 10 s, annealing at a target-specific annealing temperature and time followed by elongation at 72°C for a specific duration. S1 Table provides information regarding primer sequences, annealing temperatures, target genes, fragment sizes and number of PCR cycles conducted.

DNA from pieces of liver tissue suspected to be affected by alveolar echinococcosis were further analyzed at the Institute of Parasitology, University of Zürich, Switzerland) by a *E*. *multilocularis*-specific PCR as described by Stieger *et al*. [37].

PCR products were analyzed on agarose gels run in parallel to gene ruler size markers (Thermo Scientific). Positive samples were subjected to sequencing at either GATC Biotech (Koblenz) or LGC Genomics (Berlin). Sequences were compared to previously deposited entries in NCBI GenBank using Blastn software [38].

### Statistical analyses

Prevalence rates and 95% confidence intervals (CI) as Wilson score intervals were calculated with the propCI function in the R package “prevalence”. Pairwise differences in prevalence between various species or between individual sites were analyzed using the mid-P exact test [39] as implemented in the R package epitools.

Logistic regression analysis was performed using the “glm” function in R to estimate the influence of factorial variables rodent species or genus, study location, sex and age class (juvenile vs. adult). An interaction between sex and age class was also considered. Significance of individual levels of a factorial variable was determined via the t test statistic implemented in “glm”. The Akaike information criterion (AIC) was used to compare models and the “drop1” function in R was further used to identify variables that could be excluded from models. In addition, likelihood ratio tests (LRTs) were used to compare nested models (e.g. a model including only the variable species with a model containing the variables species and location). This test was also used for comparison of models with the null model (only intercept) and the fully parameterized model. For models with more than one variable (and variables with more than two levels) Wald tests were conducted to identify whether there was an overall significant influence of the variables. For categorical variables with only two levels, the results of the Wald test and the t test already implemented in the “glm” function are identical. In order to identify points with a particularly strong influence on the overall model, influence points were calculated as delta deviance (ΔD_i_) values. In this context, the ΔD_i_ statistics determines the change in the deviance goodness-of-fit statistic of the logistic regression model if a particular data point is omitted from the model. This ΔD_i_ was calculated from deviance residuals (d_i_), pearson residuals (r_i_) and the leverage values obtained from the diagonal of the hat matrix (h_ii_) according to ΔD_i_ = d_i_^2^ + r_i_^2^×h_ii_/(1-h_ii_). In R, d_i_, r_i_ and h_ii_ were obtained from the model using the commands residuals (model), residuals (model, type “pearson”) and influence (model)$hat, respectively. Finally, pseudo-R^2^ values according to McFadden were determined.

Non-metric multidimensional scaling (NMDS) and cluster analysis were performed to detect overall patterns in the data. For this purpose, every trapping week was considered to be a single data point. Two distinct datasets were then analyzed. In the first, the number of animals per rodent species was listed for every trapping week. In the second, the number of rodents positive for one of the parasites (ascarids, *F*. *glareoli*, *T*. *gondii*, *M*. *litteratus*, *T*. *martis*) found in the trapping week was used. For the latter, rodents were considered positive for ascarids if the *T*. *canis* ELISA was positive or if any ascarid species had been detected by PCR. Both analysis were then conducted with identical methods. Initially, a dissimilarity matrix was calculated using a Bray-Curtis distance as implemented in the vegdist function (vegan 2.4–1 R package) including a double Wisconsin standardization. Then, NMDS was performed and plotted using metaMDS from the same package with default parameters. PERMANOVA (adonis function from the vegan package) were used to determine if there are differences in rodent or parasite communities among study sites or between urban and periurban sites. The minimum number of different clusters in the dissimilarity matrix for k-means clustering was identified using NbClust 3.0 and the Gap criterion [40]. Using this minimum number of clusters the k-means function with the Hartigan-Wong algorithm and 10 random starting points was used to assign every data point to a cluster. Finally, pairwise mid-p exact tests were used to identify if a rodent or parasite species was significantly more often represented in one of the clusters than in the others. All p-values were corrected for multiple testing using the Holm correction as implemented in p.adjust.

## Results

### Rodent species captured at the four study sites

In total, 257 rodents were captured and included in the study. None of the rodents trapped belonged to the genus *Rattus*. Animals that were released were chosen from the numbered traps according to random matrices. Six different species were trapped with *A*. *flavicollis* (n = 82) being the most frequent one followed by *A*. *agrarius* (n = 78), *M*. *glareolus* (n = 59) and low to very low numbers of *A*. *sylvaticus* (n = 25), *M*. *arvalis* (n = 11) and *Microtus agrestis* (n = 2). However, the species composition was very different for the various locations (Table 1). For instance, *A*. *sylvaticus* was only found in Moabit and was almost the only species in this location (25 out of 26 animals). *M*. *glareolus* was found predominantly in the peri-urban sites Tegel (n = 44) and Gatow (n = 14) but was virtually absent in Moabit (n = 1) and absent in Steglitz.

**Table 1:** Rodent species trapped at the different study sites and included in the study.

	Study location				
	Gatow	Tegel	Moabit	Steglitz	Total
	n = 91	n = 36	n = 26	n = 104	n = 257
	Number	Number	Number	Number	Number
	Frequency^a^	Frequency^a^	Frequency^a^	Frequency^a^	Frequency
Rodent species	(95% CI^b^)	(95% CI^b^)	(95% CI^b^)	(95% CI^b^)	(95% CI^b^)
*A*.*flavicollis*	33	22	0/26	27/104	82/257
n = 82	36.3	61.1	0.0	26.0	31.9
	(27.1–46.5)	(44.9–75.2)	(0.0–12.9)	(18.5–35.1)	(26.5–37.8)
*A*. *sylvaticus*	0	0	25/26	0/104	25/257
n = 25	0.0	0.0	96.2	0.0	9.2
	(0.0–4.1)	(0–9.6)	(81.1–99.3)	(0–4.4%)	(6.7–14.0)
*A*. *agrarius*	5	0	0/26	73/104	78/257
n = 78	5.5	0.0	0.0	70.2	30.4
	(2.4–12.2)	(0–9.6)	(0.0–12.9)	(60.8–78.1)	(25.1–36.2)
*M*. *glareolus*	44	14	1/26	0/104	59/257
n = 59	48.4	38.9	3.9	0.0	22.6
	(38.4–58.5)	(24.8–55.1)	(0.7–18.9%)	(0–4.4%)	18.2–28.5%
*M*. *arvalis*	7	0	0/26	4/104	11/257
n = 11	7.7	0.0	0.0	3.9	4.3
	(3.8–15.0)	(0–9.6)	(0–12.9)	(1.5–9.5)	(2.4–4.5)
M. agrestis	2	0	0/26	0/104	11/257
n = 2	2.2	0.0	0.0%	0.0%	4.3%
	(0.6–7.7)	(0–9.6)	(0–12.9%)	(0.0–4.4%)	2.4–4.5%
Total	91	36	104/257	104/257	2/257
n = 257	35.4	14.0	40.5%	40.5%	0.8%
	(29.8–41.4)	(10.3–18.8)	(34.7–46.6%)	(34.7–46.6%)	0.2–2.8%

Values in cells combining study locations with rodent species (*Apodemus flavicollis*, *Apodemus sylvaticus*, *Apodemus agrarius*, *Myodes glareolus*, *Microtus arvalis*, *Microtus agrestis*) describe apparent frequency and 95% confidence intervals of rodent species in this particular location. In the “Total” column, the number of rodents trapped in this study location to the total number of rodents trapped. In the row “Total”, the number of rodents trapped from this species is considered in relation to the total number of rodents trapped.

^a^ Frequency at the particular study site

^b^ 95% confidence interval.

### Detection of Coccidia by PCR

Initially, DNA isolated from rodent brains was analyzed using an ITS-1 primer pair with broad specificity for all Coccidia, and it was positive for 34 animals. Sequencing revealed the presence of *Frenkelia glareoli* in all of these samples. It was detected in the present study not only in *M*. *glareolus* but also in a few *A*. *flavicollis* and one *M*. *agrestis*. S2–S7 Tables provide detailed data for Coccidia (and ascarids) for all host species, different age classes, sexes and study locations. Table 2 summarizes the results for Coccidia. Although most *F*. *glareoli* positive animals were found in the forests in Gatow and Tegel, the parasite also occurred rarely in the park in Steglitz, whereas the parasite was not found in the backyard in Moabit. Logistic regression analysis was conducted to determine the effects of rodent species or genus, location, sex and age group on the probability of detecting *F*. *glareoli*. Alternative models were calculated that either included the variable genus or the variable species but not both. Since age class and sex neither had a significant effect nor improved the statistical model in terms of a decreasing AIC, only models containing the explanatory variables species or genus and location are presented (S8 Table). As a single variable, only the species *M*. *glareolus* (when all rodent species were considered) or the genus *Myodes* (when only genera were considered) significantly increased the odds to be positive for *F*. *glareoli* (p < 0.001) (Models 1 and 2 in S8 Table). The inclusion of the variable location increased the AIC in the context of the variable species (Model 3 S8 Table) but decreased it when the variable genus was used. Due to the small number of animals in these groups, 95% CI were very wide for the genus *Microtus* (or *M*. *agrestis* and *M*. *arvalis*), for *A*. *sylvaticus* and for Moabit. The model involving species and location, further suffers from the huge influence of a single observation on the estimated parameters, i.e. the only *M*. *glareolus* trapped in Moabit and negative for *F*. *glareoli*. This was much better with the model involving only the rodent species. Odds ratios with 95% CI for *M*. *glareolus* and *A*. *agrarius* in the simple model including only species are shown in Fig 1A. Although the 95% CI for *A*. *agrarius* does not include an odds ratio of 1, the factor was not considered to be significant in the logistic regression analysis. *A*. *sylvaticus* and both *Microtus* species were omitted from the figure due to the very wide 95% CI. Only for *M*. *glareolus* the age of the animals was considered in more detail. Comparison of eye lens dry weight between animals negative and positive for *F*. *glareoli* revealed significantly higher values for positive animals (Fig 1B). In conclusion, the age of *F*. *glareoli* positive animals is significantly higher than the age of negative animals. Logistic regression for presence of *F*. *glareoli* in *M*. *glareolus* using only dry eye lens weight as explanatory variable also revealed a positive effect of increased eye lens weight (p = 0.01, odds ratio 2.81, 95% CI 1.27–7.67). Thus, for a 1 mg increase in dry eye lens weight the odds of being positive for *F*. *glareoli* increased by 2.81 fold.

**Table 2:** Prevalence rates of Coccidia in small rodents.

	Prevalence [%](95% CI^a^)	
Rodent species (n)	*Frenkelia glareolus*	*Toxoplasma gondii*
*Apodemus flavicollis* (82)	8.54 (4.2–16.6)	2.22 (0.1–8.2)
*Apodemus sylvaticus* (25)	0.00 (0–9.8)	8.00 (0.6–7.7)
*Apodemus agrarius* (78)	12.99 (2.3–7.0)	5.13 (2.0–12.5)
*Myodes glareolus* (59)	55.93 (43.3–67.8)	0 (0–4.4)
*Microtus agrestis* (2)	0 (0–57.5)	50 (9.1–90.5)
*Microtus arvalis* (11)	0 (0–19.7)	27.3 (9.7–56.6)
*Apodemus* spp. (185)	4.32 (2.2–8.3)	4.32 (2.2–8.3)
*Myodes* spp. (59)	55.93 (43.3–67.8)	0 (0–4.4)
*Microtus* spp. (13)	0 (0–17.2)	30.77 (12.7–57.6)
All (257)	13.23 (9.62–17.92)	4.66 (2.69–7.98)

^a^ 95% confidence interval.

Since the PCR with primers specific for all Coccidia did not detect any *T*. *gondii* infected animals, a second PCR was performed using a primer pair targeting a *T*. *gondii*-specific repetitive element in order to increase the sensitivity. This assay detected 12 *T*. *gondii* positive animals (4.7%) belonging to all species with exception of *M*. *glareolus* and coming from all four study locations (Table 2). The logistic regression models summarized in S9 Table include the variables genus and location since the variables sex and age class increased the AIC and no significant differences could be detected when species was combined with location due to the low number of positive animals for individual species. Odds ratios for the models including only the variable genus (with the lowest AIC) and with genus and location are shown in Fig 1C and 1D. McFadden pseudo-R^2^ values were low for both models (0.15–0.18) and for the model assuming only an effect of the genus. Calculation of influence points revealed that the *Apodemus* mice positive for *T*. *gondii* had the strongest effects on estimation of parameters but influence points were below 7 for all observations. For *Microtus* spp. the chance to be positive for *T*. *gondii* was increased by approximately 9.8-fold (p<0.001) compared with *Apodemus*. For *Myodes*, the odds ratio could not be calculated reliably by logistic regression since no positive animals were detected. Mid-p exact tests revealed that the difference in *T*. *gondii* prevalence rates between *Myodes* and *Microtus* were significant (p < 0.001) while the difference between *Myodes* and *Apodemus* was not significant (p = 0.11). If the genus and the study location were both considered as explanatory variables, the odds ratio for *Microtus* to be positive in comparison with *Apodemus* even increased from 9.8 to approximately 20. A single, *T*. *gondii* positive *Apodemus* trapped in Gatow had an influence point of 8.98, all other observations had influence points below 7, most of them much lower.

### Detection and molecular identification of metacestodes

In the body cavity of six rodents (three *M*. *glareolus*, one *A*. *agrarius*, one *A*. *flavicollis* and one *M*. *arvalis*), tetrathyridia larvae were found. Two of the animals were from Tegel, two from Steglitz and two from Gatow. An ITS-2 PCR followed by sequencing identified all these larvae as *M*. *litteratus*.

Taeniid metacestodes or tissue alterations that were suspected to be lesions due to the presence of *E*. *multilocularis* were subjected to a PCR with primers amplifying the ITS-2 or a sub-fragment of the mitochondrial small rRNA gene of plathelminthes or by an *E*. *multilocularis*-specific PCR. Of the six liver lesions suspected to be *E*. *multilocularis*, none was positive for flatworm DNA in any of the PCRs.

Taeniid metacestodes were found in five animals, two *M*. *glareolus* (from Gatow and Tegel), one *A*. *agrarius* (from Steglitz) and two *A*. *flavicollis* (from Steglitz and Gatow). Sequencing of the ITS-2 (accession-no. KT943418- KT943421) revealed 74% identity to *Taenia crassiceps*, most likely representing a closely related tapeworm whose ITS-2 sequence has not been deposited in GenBank yet. The ITS sequences were very similar to each other (94–96% identity) but there were also 8 gaps of 3–15 bp in the alignments. Amplification and sequencing of a mitochondrial 12S rRNA gene from three samples (accession-no. KT943414- KT943416) revealed 99% identity between the three samples and two *Taenia martis* entries in GenBank. Therefore, the metacestodes were considered to belong to this species. Finally, in the liver of a *M*. *glareolus* from Tegel at least 10 specimens of a third metacestode species were found which was microscopically identified as *Cladotaenia globifera* (Paruterinidae). ITS-2 and 12S RNA gene sequences for these metacestodes were determined and deposited in GenBank under the accession no. KT943422 and KT943417, respectively.

### Ascarid detection by PCR and ELISA

For the detection of ascarids by PCR, DNA from skeletal muscle and brain tissue of all 257 rodents, were used as template, and animals were considered positive if one of the PCRs was positive. Eight animals (three *M*. *glareolus*, two *A*. *flavicollis*, two *A*. *agrarius*, and one *A*. *sylvaticus*) were positive for *T*. *canis*: six in the PCR using muscle and two (one *A*. *flavicollis*, one *A*. *agrarius*) in the PCR using brain DNA but none of them in both PCRs. This corresponds to an estimated prevalence of 3.1% (95% CI: 1.6–6.0%) for all rodent species together. In four (two *A*. *agrarius*, one *A*. *flavicollis* and one *A*. *sylvaticus*) and one (*A*. *flavicollis*) of the skeletal muscle DNAs *T*. *cati* and *Parascaris* sp. DNA were detected, respectively. This corresponds to prevalence rates of 1.6% (95% CI: 0.6–3.9%) and 0.4% (95% CI: 0.1–2.2%). ELISA was more sensitive than PCR and detected antibodies against *T*. *canis* in 34 animals (prevalence 14.2%; 95% CI: 10.3–19.1%) (see Table 3 for individual rodent species and study locations). Sera from seven out of eight animals positive for *T*. *canis* in PCR were also positive for *Toxocara* in ELISA while none of the *T*. *cati* or *Parascaris* spp. positive animals reacted in the *T*. *canis* ES antigen ELISA.

**Table 3:** Prevalence rates of antibodies against *Toxocara canis* excretory-secretory antigen.

	Study location				
	Gatow	Tegel	Moabit	Steglitz	Total
	Number^a^	Number^a^	Number^a^	Number^a^	Number^a^
	Frequency [%]	Frequency [%]	Frequency [%]	Frequency [%]	Frequency [%]
Rodent species	(95% CI^b^)	(95% CI^b^)	(95% CI^b^)	(95% CI^b^)	(95% CI^b^)
*A*.*flavicollis*	33	21	0	23	77
	0	4.76	na	0	1.3
	(0–10.4)	(0.8–22.7)	na	(0–14.3)	(0.2–7.0)
*A*. *sylvaticus*	0	0	25	0	25
	na^c^	Na	28.0	na	28.0
	na	Na	(14.3–47.6)	na	(14.3–47.6)
*A*. *agrarius*	5	0	0	67	72
	60.0	Na	na	28.4	33.3
	(23.1–88.2)	Na	na	(19.0–40.1)	(23.5–44.8)
*M*. *glareolus*	43	12	1	0	56
	0	16.7	0	na	3.6
	(0–8.2)	(4.7–44.8)	(0–79.3)	na	(1.0–12.1)
*M*. *arvalis*	2	0	0	0	2
	0	Na	na	na	0
	(0.0–65.8)	na	na	na	(0–65.8
M. agrestis	5	0	0	3	8
	0	na	na	0	0
	(0.0–43.5)	na	na	(0.0–63.2)	0–32.4
Total	88	33	26	93	240
	3.4	9.1	26.9	20.4	14.2
	(1.2–9.5)	(3.1–23.6)	(13.7–46.1)	(13.7–29.7)	(10.3–19.1)

Seroprevalence is reported separately for all rodent species (*Apodemus flavicollis*, *Apodemus sylvaticus*, *Apodemus agrarius*, *Myodes glareolus*, *Microtus arvalis*, *Microtus agrestis*) and study sites.

^a^The total number of examined animals is lower than in Table 1 since serum was not obtained from all rodents.

^b^95% confidence interval.

^c^na, not available.

**Fig 2:**
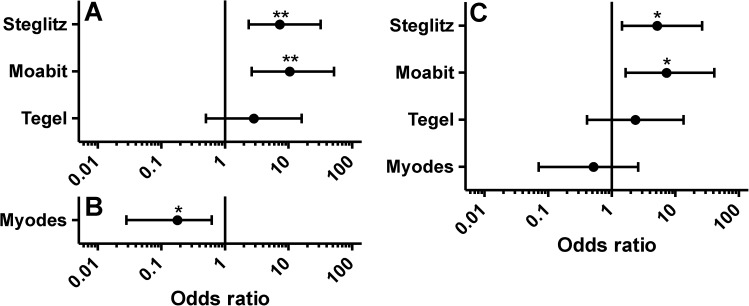
Odds ratios with 95% confidence intervals for variables determining the risk for detection of antibodies against *Toxocara canis*. Logistic regression was conducted using the variables study location (A), rodent genus (B) and a combination of both (C). Reference categories were *Apodemus* for genus and Gatow for study location. The odds ratios for the genus *Microtus* were 1.1×10^−7^ in both models in (B) and (C) but are not presented since antibodies against *T*. *canis* were not found in any sample from this genus and the very wide 95% confidence intervals. *, p < 0.05; **, p < 0.01.

Logistic regression analysis was performed using the *T*. *canis* ES antigen ELISA data since so few positive animals were found by PCR. Again, the variables rodent species or genus, location, sex and age group were initially considered to explain the distribution of positive animals. However, inclusion of age severely increased the AIC and was therefore not further considered. Models using the explanatory variable rodent species or genus with or without location or only location are summarized in S1 Information and S10 Table. In some models, inclusion of the sex slightly decreased the AIC but LRT comparing nested models did not show a significant improvement of the models and sex was therefore not included. When considered separately, the variables species and location had a significant influence on the odds to be positive for *T*. *canis* with *A*. *sylvaticus* and *A*. *agrarius* having about 3.5fold higher odds (p < 0.001 and p < 0.01, respectively) than *A*. *flavicollis* and with Moabit and Steglitz having odds ratios of 10.4 and 7.3 (p<0.01) compared to Gatow (Fig 2A). If only the variable genus was included in the model, the AIC of the model was 183.23 and *Myodes* had a significantly lower odds (decreased by approximately 5.6 fold) than *Apodemus* (p = 0.02) (Fig 2B). Inclusion of the study site did improve the AIC (180.22) but in this combination only the parameters Moabit and Steglitz had a significant influence on the odds (Fig 2C) suggesting that the study location is more important than the rodent genus although both variables are strongly collinear. However, Wald tests for the significance of the variables study location and rodent genus revealed non-significant influences on the probability to detect *T*. *canis* antibodies (i.e. 0.75 for genus and 0.074 for study site). The pseudo-R^2^ for this model is also quite poor (0.11) and a LRT comparing these two models revealed that the model with the parameters genus and study location was not significantly better than the model containing only the parameter location (p = 0.28). In contrast, this model was significantly better than the model considering only the variable genus (p = 0.011).

### Observed co-infections

In a few cases, co-infections were detected in the study population. One *M*. *glareolus* from Tegel was heavily triple infected and hosted 512 *M*. *litteratus* metacestodes, one *T*. *martis* and more than 10 *Cladotaenia globifera* (Table 4). Among the animals positive for *T*. *canis* in the ELISA, two and one were also positive by PCR for *F*. *glareoli* or *T*. *gondii*, respectively. Another two animals were positive for both, *M*. *litteratus* and *F*. *glareoli* and one animals was positive for *T*. *cati* and *M*. *litteratus*. One of the *T*. *martis* positive animals was also positive for *F*. *glareoli*. Details are provided in Table 4.

**Table 4:** Parasite co-infections found in examined mice (n = 257).

Parasite combination	Number of rodents
1. *Mesocestoides litteratus* larvae *2. Taenia martis* larvae 3. *Cladotaenia globifera* larvae	1
1. *Toxocara canis* ELISA 2. *Toxoplasma gondii* PCR	1
1. *Mesocestoides litteratus* larvae *2. Frenkelia glareoli* PCR	2
1. *Toxocara canis* ELISA 2. *Frenkelia glareoli* PCR	2
1. *Mesocestoides litteratus* larvae 2. *Toxocara cati* PCR	1
1. *Taenia martis* larvae 2. *Frenkelia glareoli* PCR	1

### Non-metric multi-dimensional scaling and cluster analyses

**Fig 3:**
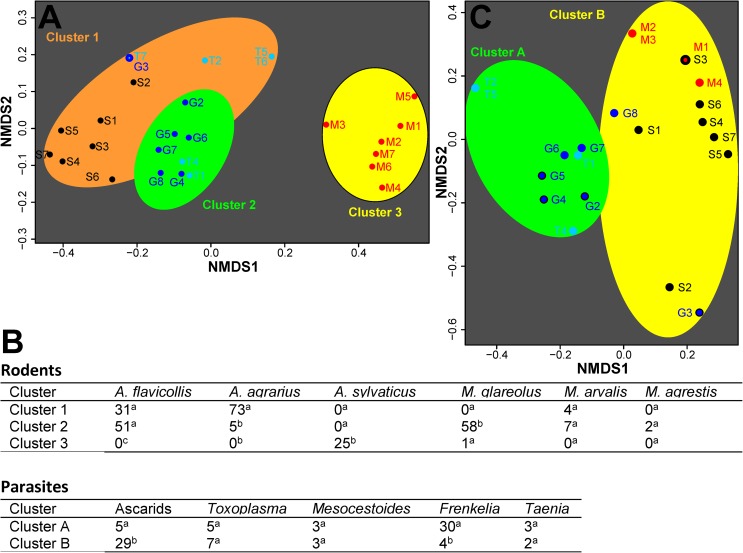
Identification of patterns using non-metric multidimensional scaling (NMDS) and k-means cluster analysis. NMDS plots show similarity for trapped rodent species (A) or parasite-positive rodents (C) for the different trapping sites (G, Gatow, M, Moabit, S, Steglitz, T, Tegel) and trapping blocks (1–8). Rodents were considered to be positive for ascarids if the *T*. *canis* ELISA was positive or any ascarid species was detected by PCR. For every trapping week, the number of rodents of a particular species or the number positive for a particular parasite was used to calculate a Bray-Curtis dissimilarity matrix followed by NMDS. The matrix was used to identify the minimum number of k-means clusters which were indicated on the NMDS plots using ellipses of different colors. (B) Clusters were analyzed for differences in the proportions of particular rodent or parasite species (species relative to total number in that cluster) using mid-P exact tests. Numbers with different indices in the same column indicate significant differences (p<0.01) while identical indices indicate non-significant differences (p>0.05).

Fig 3A shows a plot of NMDS for the rodent data. PERMANOVA revealed significant differences between individual study sites as well as between urban and peri-urban sites (p < 0.001). The minimum number of k-means clusters identified in the data was 3 and clusters were marked in the NMDS plot in Fig 3A using differently colored ellipses. Cluster 1 contains all data points from Steglitz as well as a few data points from Gatow and Tegel. This cluster is characterized by a significantly higher abundance of *A*. *agrarius* (p<0.05 vs. cluster 2 and 3 in a mid-P exact test) and the absence of *M*. *glareolus* (Fig 3B). Cluster 2 contains most of the data points from Gatow and Tegel. NMDS shows that this cluster is relatively close to and thus similar to cluster 1 (Fig 3A) but it is discriminated from the latter by a high abundance of *M*. *glareolus* (Fig 3B) (p<0.01 vs. cluster 1 and 3). Both cluster 1 and cluster 2 share a high abundance of *A*. *flavicollis* (Fig 3B) (p<0.01 vs. cluster 3). Cluster 3 contains all data from Moabit and it has a large distance to the other two clusters in the NMDS plot (Fig 3A). The cluster is characterized by the fact that *A*. *sylvaticus* was exclusively found here (p<0.01 vs. cluster 1 and 2) and was nearly the only rodent species that was identified here (Fig 3B).

The complementary analysis using the parasite data is shown in Fig 3C. Again, parasite diversity differed between individual study sites as well as urban and peri-urban sites (PERMANOVA, p < 0.001). In comparison with the rodent data, only two clusters could be identified. The first cluster A contains most of the peri-urban data from Gatow and Tegel with two exceptions from Gatow. This cluster is characterized by a low abundance of ascarids and a high abundance of *F*. *glareoli* (both p<0.01 in a mid-P exact test vs. cluster B). The second cluster B contains all the data from the urban sites Moabit and Steglitz plus the remaining two data points from Gatow. This cluster reveals high abundance of ascarids and low abundance of *F*. *glareoli*. The other parasites (*T*. *gondii*, *M*. *litteratus* and *T*. *martis*) were found only in small numbers but also in both clusters and did not contribute to this pattern. In this cluster, two points (Gatow 3 and Steglitz 2) can be considered as outliers. In these trapping weeks neither ascarids nor *F*. *glareoli* were identified. The only parasites that were found in these trapping weeks were tapeworms. The other data point from Gatow (G8) which was assigned to the cluster B represents a trapping week in which two of the five ascarid positive rodents from periurban sites were found. It also contributes two of the four *F*. *glareoli* positive rodents to cluster B. The other two were found in Steglitz in trapping action 1 (S1), a dataset which also contains 3 ascarid positive rodents and which is located at a very similar position in the NMDS plot as G8. Datasets G8 and S1 can be considered to have an intermediate position between both clusters.

## Discussion

Rodent-borne zoonotic diseases impose a substantial health threat to pet animals and humans [4, 5]. Parasites of cats and dogs with rodent reservoirs are associated with particular additional risks due to the close contact between companion animals and their owners. From this point of view it can be viewed positive that the parasite most frequently identified in this study uses birds of prey as definitive hosts and is of no relevance for human or companion animal health. Nevertheless, there was a considerable number of rodents positive for important zoonotic pathogens including *T*. *gondii* and *Toxocara* spp. Moreover, the fact that the latter was more often identified in urban than peri-urban settings suggests that anthropogenic influences can facilitate transmission of this parasite.

The assays performed to detect Coccidia were planned with *T*. *gondii* as the focus parasite. Since cysts of these parasites are predominantly found in neuronal tissues, only DNA isolated from brain tissue was analyzed and *Sarcocystis* species were largely excluded by this study design. The parasite with the highest prevalence found in this study, *F*. *glareoli*, is known to circulate between bank voles and common buzzards (*B*. *buteo*). In the present study it was not only found in bank voles, its most important intermediate host, but also in a few *Apodemus* mice. This is not too surprising since *Frenkelia* species are known to be not strictly specific regarding their intermediate hosts. In a large study examining brains of 1760 small rodents from the Czech Republic microscopically, Svobodova *et al*. [41] identified one *F*. *glareoli* positive *Apodemus* (n = 654). However, the vast majority of brain cysts in *Apodemus* spp. represented *Frenkelia microti*, which also infects common buzzards as definitive hosts. *F*. *microti* was also found in three *M*. *glareolus* (n = 486). In contrast, Fichet-Calvet *et al*. [42] did not detect *F*. *microti* in any of 311 *Apodemus* spp. collected in France, whereas four were positive for *F*. *glareoli*. *F*. *microti* was most prevalent in *M*. *arvalis* and *M*. *agrestis* but it was also found in three *M*. *glareolus* (n = 367). In summary, *F*. *glareoli* showed a much higher prevalence in *M*. *glareolus* as intermediate host than in other rodents in all three studies. However, its occurrence in other species varied considerably between studies. This variation is presumably due to unrecognized differences between study sites. The fact that no *F*. *microti* were detected in rodents collected in Berlin might be explainable by the low number of *Microtus* spp. that were trapped. Differences in the methodology (microscopic examination vs. PCR) also cannot be excluded to contribute to this difference, in particular since no ITS-2 sequence has been deposited in GenBank for *F*. *microti*.

The PCR targeting a *T*. *gondii*-specific repetitive element identified a low number of 12 positive samples with significantly increased odds of *Microtus* spp. to be positive compared to *Apodemus*. However, due to the low number of *Microtus* animals included in the study, the validity of this finding should nevertheless be interpreted with care when comparing with the results of other studies. Herrmann *et al*. [43] have recently analyzed rodents from the Western part of Brandenburg, close to the border with Saxony-Anhalt, less than 90 km away from the nearest trapping site in the present study (Gatow). Among the 72 rodents tested in their study (69 *M*. *arvalis*), none was positive in an immuno-blot assay using purified native *Tg*SAG-1 surface protein as target or in the same PCR used here. The lower prevalence observed by PCR might be due to important differences in the ecological systems the rodents were obtained from (rural area with low population density vs. urban or peri-urban area) but the use of different sources of DNA (heart vs. brain tissue) presumably will also contribute to such differences. Very recently, *T*. *gondii* and *N*. *caninum* were found to be present in house mice (*Mus musculus*) at the Czech-German border in low prevalence rates of 0.6% and 3.6%, respectively [44]. In the UK, 3% of the house mice were found to be positive for *N*. *caninum* while 53% contained *T*. *gondii* DNA and in rats, prevalence rates of 2% and 42.2% were reported, respectively [45]. Meerburg et al. [46] analyzed more diverse species of rodents and found *T*. *gondii* exclusively in house mice and bank voles while *N*. *caninum* was also found in wood and harvest mice. However, the number of animals investigated per individual host species was low (between three and 24) with the exception of house mice (n = 78). Therefore, quantitative interpretation of these data is difficult.

Herrmann *et al*. [43] compared PCR analysis with immunoblot analysis using recombinant TgSAG-1, and analyses of samples from foxes revealed that the immuno-blot assays were considerably more sensitive than the PCR. Using a commercially available tachyzoite antigen preparation as target, Reperant *et al*. [47] had found 2.5 and 5% of *A*. *flavicollis* and *M*. *arvalis* captured in the canton Geneva (Switzerland) to be positive for *T*. *gondii*, respectively. Over all host species, seroprevalence rates varied considerably between study sites (2.9–17.2%). Due to considerations regarding animal welfare, serum samples had to be collected after euthanasia in the present study, resulting in smaller volumes of serum. This fact did not allow performing immuno-blot analyses since only small amounts of serum were left after the ELISA for *T*. *canis* had been performed. All attempts to obtain reliable results with the ELISA described by Reperant *et al*. [47] unfortunately failed. Therefore, no comparison to results reported in the literature is possible. However, low prevalence rates for *T*. *gondii* were also observed for *M*. *arvalis* (0.7%) and *A*. *terrestris* (4.7%) in a study in Vorarlberg (Austria) [48], although the PCR used was presumably less sensitive since it does not target a repetitive element. Using a nested PCR amplifying a fragment of a single-copy gene (SAG-1), much higher prevalence rates were described in the UK (Manchester) in *M*. *musculus* (53%) and *Rattus norvegicus* (42.2%) in an urban area [45]. Using the same detection approach, a comparably high prevalence of 40.8% was reported for *A*. *sylvaticus* in an area with low cat density suggesting that the contribution of vertical transmission to the epidemiological status of a rodent population may not be negligible [49].

Regarding cestode larvae, it is most important to emphasize that no *E*. *multilocularis* were detected in any of the samples. Since Staubach *et al*. [50] reported an *E*. *multilocularis* prevalence of 2.4% in foxes in an area of Brandenburg only approximately 60–100 km north-west of Berlin, it is well known that this parasite is endemic in the surrounding area. Remarkable increases in *E*. *multilocularis* prevalence rates in foxes have been reported for Saxony-Anhalt (13.6% between 1998 and 2005 to 23.4% from 2006 to 2010) [51] and Thuringia (11.9 to 42% between 1990 and 2005) [52]. The fact that no *E*. *multilocularis* were detected in intermediate rodent hosts in the urban area of Berlin suggests that it is either absent or only present in low prevalence. This might appear surprising given the high fox densities but is at the same time of course good news for public health. Unfortunately, no published data is available regarding *E*. *multilocularis* prevalence in foxes in Berlin. However, this is in agreement with information provided by local authorities involved in fox monitoring. Due to the rapid increases observed in other eastern German states, dense monitoring schemes should nevertheless be considered to determine future changes since the currently only spotted distribution of this parasite might soon lead to a generally endemic situation. Moreover, it should be emphasized that it is apparently difficult to detect metacestodes in rodents even in regions with high prevalence of *E*. *multilocularis* [53].

The cestodes *T*. *martis*, which uses mustelids as definitive hosts, and *M*. *litteratus*, a parasite of foxes and rarely also of dogs and cats, are known to be widely distributed in central Europe and to use rodents as intermediate hosts. *M*. *litteratus* has previously been shown to be highly endemic in central Europe. In particular, it has been described to occur in more than 40% of red foxes in the Slovak Republik [54] and larval stages have been found in rodents of the species *A*. *agrarius* and *M*. *glareolus* [55]. *T*. *martis* has recently been considered to be an emerging infectious disease since it has been recognized to rarely cause infections of the eye and the brain in humans [56, 57] and to cause severe to fatal infections in non-human primates [58, 59]. The ITS-2 sequences obtained from four specimen here show a remarkably high diversity. Further molecular studies are required to determine if these genotypes prefer different intermediate or definitive hosts. More remarkable than the fact that these two parasites were found, is the fact that larval stages of typical cestodes of dogs and cats such as *T*. *crassiceps* and *Hydatigera taeniaeformis* were not found at all which is in contrast to the results obtained by Reperant et al. [47].

Using PCR, three ascarid species were detected, one of them surprisingly being *Parascaris*, the horse roundworm. Since its definitive host is herbivore, paratenic hosts should not be of any epidemiological importance but apparently *Parascaris* larvae are able to migrate in such dead-end hosts. Expectedly, all *T*. *cati* were detected in muscle tissue but the fact that more muscle than brain samples were positive for *T*. *canis* is in contrast to the reported tissue tropism in laboratory mice [60]. In the definitive host, hypobiotic *T*. *canis* larvae are also predominantly found in muscles [6] suggesting that the distribution in house mice and *Apodemus* and *Myodes* might differ as already known for rats and gerbils [60]. Due to the low number of positive animals in the present study and the fact that none of them was positive in both, brain and muscle, these results can only be a first hint that wild rodents might differ in larval migration patterns from commonly used laboratory models and experimental infections would be needed to address this aspects more thoroughly. It is furthermore noteworthy to mention that none of the samples was positive for *Baylisascaris procyonis*, the racoon roundworm, which can cause severe neurological disease in humans [61].

ELISAs using *T*. *canis* ES antigen have been used several times to determine seroprevalence in rodents and definitive hosts such as foxes. In general, this ELISA is not considered to be very specific. Despite the fact that it shows no or little cross-reactivity with *Ascaris suum* and *Toxascaris leonina* [62, 63], some level of cross-reactivity between *T*. *canis* and *T*. *cati* can be expected and cross-reactivity to *Toxocara vitulorum* and *B*. *procyonis* has to the knowledge of the authors never been addressed. However, the fact that none of the four mice positive for *T*. *cati* was detected as positive in the ELISA while 7 out of 8 *T*. *canis* PCR-positive mice were also positive in the ELISA suggests a certain specificity. Recent attempts comparing recombinant ES antigens from both *Toxocara* species also showed considerable cross-reactivity [64, 65] and initial experiments using monoclonal antibodies raised against *T*. *canis* antigens in a capture ELISA to detect circulating larval antigens showed high specificity (100%) but only low sensitivity (31%) to detect human toxocarosis [66]. The overall seroprevalence of 14.2% observed in Berlin is quite high in comparison to what has been described in other studies in central Europe. For instance, Reperant *et al*. [47] found 6.5% positive animals in Switzerland. In two studies from the Slovak Republic, 6.4 and 7.7% seroprevalence rates were reported [28, 67]. The distribution of seropositive samples regarding host species was very different between these studies. While in Switzerland similar prevalence rates (approximately 5–7%) were observed for all species trapped, Antolova *et al*. [67] found a significantly higher prevalence in *A*. *agrarius* (15.9%) compared with all other rodents species (0–3.5%). Recently, Antolova *et al*. [28] detected high prevalence rates in *A*. *agrarius* (10.9%) and *Mus spicilegus* (11.2%) while rates of seropositivity were much lower in *A*. *flavicollis* (4.2%) and *M*. *glareolus* (3.6%). They found that granivores (*Apodemus*, *Mus*, *Micromys*) or insectivores had a higher risk of being exposed to *Toxocara* than herbivorous species (*Myodes*, *Microtus*). In a previous study, the same group had shown that prevalence in rural areas was almost half of that observed in suburban environments [67]. In the present study, very large differences in species-specific prevalence rates were recorded (range 0–33%). However, these differences were not significant in logistic regression analysis due to the strong co-linearity of the factors study location and host species. Considering only the host genus, rodents from the locations in Moabit and Steglitz had significantly increased odds of being positive for *T*. *canis* in the ELISA. Similar to what was observed in Berlin, two previous studies detected a higher chance to be seropositive for antibodies against *T*. *canis* ES antigens in urban compared to peri-urban or rural areas [47, 67].

NMDS and cluster analyses were used to detect overall patterns in the data. There were highly significant differences among study sites as well as between urban and peri-urban sites for both, rodent and parasite communities. More interestingly, cluster analysis identified three clusters in rodent data whereas only two clusters were identified in parasite data. Rodent data showed a relatively close proximity between three study sites and the peri-urban sites in Gatow and Tegel and the urban site in Steglitz were close together in the NMDS plot. Cluster 1 contained data from all these three sites while cluster 2 contained only data from Gatow and Tegel, i.e. all data points when *M*. *glareolus* was present. Structuring of parasite data was obviously not dominated by rodent species since this would have caused a very similar structure as observed for rodent data. In contrast, only two major clusters were observed that largely corresponded to peri-urban and urban sites. The two most frequently detected parasites/parasite groups showed highly significant differences between these clusters. While the predominantly urban cluster was characterized by a high abundance of ascarids, the peri-urban cluster revealed a high abundance of *F*. *glareoli*. The latter can be easily explained by the fact that the bank vole as most important intermediate host was virtually absent in urban trapping sites. However, differences in the abundance of the definitive host, the common buzzard, between peri-urban forests and the highly urbanized city center might also contribute to this gradient. The parasite group that was particularly typical for urban sites were ascarids. This is somewhat astonishing since most of the positive rodents were detected positive in a *T*. *canis* ELISA while the park in Steglitz was not accessible to pet dogs. However, two foxes were observed during the study period at this site demonstrating that the park is accessible to foxes. There is presumably a dense fox population in Berlin although no data have been published so far. Comparisons between urban and peri-urban sites regarding fox densities are also missing. In Zürich, however, it has already been shown that urban fox populations can have substantially higher densities than populations in surrounding periurban or rural/sylvatic areas [1, 37]. Another explanation for this significant difference might also be that ascarid eggs are extremely common in urban areas with high densities of dogs and cats. Modelling approaches identified either young dogs or stray cats as main sources of contamination with only marginal contribution from foxes [68, 69]. High numbers of eggs might lead to a spill-over of eggs even into areas where dogs have no direct access, e.g. by passive transport with surface water, soil or human shoes. The fact that one can currently only speculate regarding the driving forces for the local differences in parasite communities including important zoonotic agents such as *Toxocara* spp. particularly emphasizes the current knowledge gaps.

In conclusion, several zoonotic parasites were detected in wild rodents in Berlin even in densely populated city areas. However, *E*. *multilocularis*, probably the most dangerous zoonotic agent transmitted between foxes (as the most relevant wild carnivore) and rodents as intermediate hosts, were not detected in the present study. In a park where dogs do not have access, prevalence rates of zoonotic parasites in rodents were not lower than in the other study areas. *Toxocara* spp. was the most frequently identified zoonotic parasite in the study. Since several zoonotic parasites, causing neglected or emerging diseases, have been detected in the current study, increased surveillance is clearly warranted.

## Supporting information

S1 Table Primer sequences and PCR conditions. (DOCX)

S2 Table Parasite prevalences in *Apodemus flavicollis*. (DOCX)

S3 Table Parasite prevalences in *Apodemus sylvaticus*. (DOCX)

S4 Table Parasite prevalences in *Apodemus agrarius*. (DOCX)

S5 Table Parasite prevalences in *Myodes glareolus*. (DOCX)

S6 Table Parasite prevalences in *Microtus arvalis*. (DOCX)

S7 Table Parasite prevalences in *Microtus agrestis*. (DOCX)

S8 Table Models for logistic regression describing presence of *Frenkelia glareoli* DNA. (XLSX)

S9 Table Models for logistic regression describing presence *Toxoplasma gondii* DNA. (XLSX)

S10 Table Models for logistic regression describing presence of antibodies against *Toxocara canis*. (XLSX)

S1 Information Detailed description of models for logistic regression of seroprevalence data for *Toxocara canis*. (DOCX)

## References

[1] Šálek M, Drahníková L, Tkadlec E. Changes in home range sizes and population densities of carnivore species along the natural to urban habitat gradient. Mammal Review. 2015;45:1–14.

[2] Sutor A, Schwarz S, Conraths F. The biological potential of the raccoon dog (*Nyctereutes procyonoides*, Gray 1834) as an invasive species in Europe—new risks for disease spread? Acta Theriol. 2014;59:49–59. 10.1007/s13364-013-0138-9 PMC7097217 32226062

[3] Duscher GG, Leschnik M, Fuehrer HP, Joachim A. Wildlife reservoirs for vector-borne canine, feline and zoonotic infections in Austria. Int J Parasitol Parasites Wildl. 2015;4:88–96. 10.1016/j.ijppaw.2014.12.001 25830102 PMC4356739

[4] Meerburg BG, Singleton GR, Kijlstra A. Rodent-borne diseases and their risks for public health. Crit Rev Microbiol. 2009;35:221–70. 10.1080/10408410902989837 19548807

[5] Bordes F, Blasdell K, Morand S. Transmission ecology of rodent-borne diseases: New frontiers. Integr Zool. 2015;10:424–35. 10.1111/1749-4877.12149 26176684

[6] Schnieder T, Laabs EM, Welz C. Larval development of *Toxocara canis* in dogs. Veterinary parasitology. 2011;175:193–206. 10.1016/j.vetpar.2010.10.027 21095061

[7] Hotez PJ, Gurwith M. Europe's neglected infections of poverty. Int J Infect Dis. 2011;15:e611–9. 10.1016/j.ijid.2011.05.006 21763173

[8] Hotez PJ, Wilkins PP. Toxocariasis: America's most common neglected infection of poverty and a helminthiasis of global importance? PLoS Negl Trop Dis. 2009;3:e400 10.1371/journal.pntd.0000400 19333373 PMC2658740

[9] Hotez PJ. Neglected infections of poverty in the United States of America. PLoS Negl Trop Dis. 2008;2:e256 10.1371/journal.pntd.0000256 18575621 PMC2430531

[10] Finsterer J, Auer H. Parasitoses of the human central nervous system. J Helminthol. 2013;87:257–70. 10.1017/S0022149X12000600 23046708

[11] Ngugi AK, Bottomley C, Kleinschmidt I, Wagner RG, Kakooza-Mwesige A, Ae-Ngibise K, et al Prevalence of active convulsive epilepsy in sub-Saharan Africa and associated risk factors: cross-sectional and case-control studies. Lancet Neurol. 2013;12:253–63. 10.1016/S1474-4422(13)70003-6 23375964 PMC3581814

[12] Palmer BS. Meta-analysis of three case controlled studies and an ecological study into the link between cryptogenic epilepsy and chronic toxoplasmosis infection. Seizure. 2007;16:657–63. 10.1016/j.seizure.2007.05.010 17604653

[13] Macpherson CN. The epidemiology and public health importance of toxocariasis: a zoonosis of global importance. Int J Parasitol. 2013;43:999–1008. 10.1016/j.ijpara.2013.07.004 23954435

[14] Maenz M, Schluter D, Liesenfeld O, Schares G, Gross U, Pleyer U. Ocular toxoplasmosis past, present and new aspects of an old disease. Prog Retin Eye Res. 2014;39:77–106. 10.1016/j.preteyeres.2013.12.005 24412517

[15] Afonso C, Paixao VB, Costa RM. Chronic *Toxoplasma* infection modifies the structure and the risk of host behavior. PLoS One. 2012;7:e32489 10.1371/journal.pone.0032489 22431975 PMC3303785

[16] Chieffi PP, Aquino RT, Pasqualotti MA, Ribeiro MC, Nasello AG. Behavioral changes in *Rattus norvegicus* experimentally infected by *Toxocara canis* larvae. Rev Inst Med Trop Sao Paulo. 2010;52:243–6. 21049227 10.1590/s0036-46652010000500004

[17] Vyas A, Kim SK, Giacomini N, Boothroyd JC, Sapolsky RM. Behavioral changes induced by *Toxoplasma* infection of rodents are highly specific to aversion of cat odors. Proc Natl Acad Sci U S A. 2007;104:6442–7. 10.1073/pnas.0608310104 17404235 PMC1851063

[18] Beste C, Getzmann S, Gajewski PD, Golka K, Falkenstein M. Latent *Toxoplasma gondii* infection leads to deficits in goal-directed behavior in healthy elderly. Neurobiol Aging. 2014; 35:1037–44. 10.1016/j.neurobiolaging.2013.11.012 24315729

[19] Walsh MG, Haseeb MA. Reduced cognitive function in children with toxocariasis in a nationally representative sample of the United States. Int J Parasitol. 2012;42:1159–63. 10.1016/j.ijpara.2012.10.002 23123274

[20] Scheid R, Tina Jentzsch R, Schroeter ML. Cognitive dysfunction, urinary retention, and a lesion in the thalamus—beware of possible toxocariasis of the central nervous system. Clin Neurol Neurosurg. 2008;110:1054–7. 10.1016/j.clineuro.2008.06.014 18687519

[21] Flegr J. Effects of *Toxoplasma* on human behavior. Schizophr Bull. 2007;33:757–60. 10.1093/schbul/sbl074 17218612 PMC2526142

[22] Flegr J. Influence of latent *Toxoplasma* infection on human personality, physiology and morphology: pros and cons of the *Toxoplasma*-human model in studying the manipulation hypothesis. J Exp Biol. 2013;216:127–33. 10.1242/jeb.073635 23225875

[23] Kunz TH, Wemmer C, Hayssen V. Sex, age and reproductive conditions of mammals In: Wilson DE, Cole FR, Nichils JD, Rudran R, Foster MS, editors. Measuring and monitoring biological diversity: Standard methods for mammals Washington D.C.: Smithonian Institution Press; 1996 p. 279–90.

[24] Niethammer J, Krapp F. Handbuch der Säugetiere Europas (in German). 2 ed. Wiesbaden: Akademische Verlagsgesellschaft; 1982.

[25] Morris P. A review of mammalian age determination methods. Mammal Review. 1972;2:69–104.

[26] Ryzhikov KM, Gvozdev EV, Tokobaev MM, Shaldybin LS, Matzaberidze GV, Merkusheva IV, et al Key to the helminth parasites of rodents from the fauna of USSR. Cestodes and trematodes (in Russian). Moskva: Nauka 1978. 332 p.

[27] Schmidt S. Untersuchungen zum Vorkommen von *Capillaria hepatica* und Metazestoden der Cyclophyllida bei Wildmäusen in Deutschland (in German). Leipzig: Universität Leipzig; 2001.

[28] Antolova D, Reiterova K, Stanko M, Zalesny G, Fricova J, Dvoroznakova E. Small mammals: paratenic hosts for species of *Toxocara* in eastern Slovakia. J Helminthol. 2013;87:52–8. 10.1017/S0022149X11000848 22284742

[29] Cuellar C, Fenoy S, Guillen JL. Cross-reactions of sera from *Toxascaris leonina* and *Ascaris suum* infected mice with *Toxocara canis*, *Toxascaris leonina* and *Ascaris suum* antigens. Int J Parasitol. 1995;25(6):731–9. 7657459 10.1016/0020-7519(94)00187-s

[30] Gasser RB, Chilton NB. Characterisation of taeniid cestode species by PCR-RFLP of ITS2 ribosomal DNA. Acta Trop. 1995;59:31–40. 7785524 10.1016/0001-706x(94)00085-f

[31] Ho MS, Barr BC, Marsh AE, Anderson ML, Rowe JD, Tarantal AF, et al Identification of bovine *Neospora* parasites by PCR amplification and specific small-subunit rRNA sequence probe hybridization. J Clin Microbiol. 1996;34:1203–8. 32. 8727903 10.1128/jcm.34.5.1203-1208.1996 PMC228982

[32] Homan WL, Vercammen M, De Braekeleer J, Verschueren H. Identification of a 200- to 300-fold repetitive 529 bp DNA fragment in *Toxoplasma gondii*, and its use for diagnostic and quantitative PCR. Int J Parasitol. 2000;30:69–75. 33. 10675747 10.1016/s0020-7519(99)00170-8

[33] Ishiwata K, Shinohara A, Yagi K, Horii Y, Tsuchiya K, Nawa Y. Identification of tissue-embedded ascarid larvae by ribosomal DNA sequencing. Parasitol Res. 2004;92:50–2. 10.1007/s00436-003-1010-7 14598166

[34] Reischl U, Bretagne S, Kruger D, Ernault P, Costa JM. Comparison of two DNA targets for the diagnosis of Toxoplasmosis by real-time PCR using fluorescence resonance energy transfer hybridization probes. BMC Inf Dis. 2003;3:7. 10.1186/1471-2334-3-7 PMC156600 12729464

[35] Bowles J, Blair D, McManus DP. Genetic variants within the genus *Echinococcus* identified by mitochondrial DNA sequencing. Mol Biochem Parasitol. 1992;54:165–73. 1435857 10.1016/0166-6851(92)90109-w

[36] von Nickisch-Rosenegk M, Lucius R, Loos-Frank B. Contributions to the phylogeny of the Cyclophyllidea (Cestoda) inferred from mitochondrial 12S rDNA. J Mol Evol. 1999;48:586–96. 10198124 10.1007/pl00006501

[37] Stieger C, Hegglin D, Schwarzenbach G, Mathis A, Deplazes P. Spatial and temporal aspects of urban transmission of *Echinococcus multilocularis*. Parasitology. 2002;124(Pt 6):631–40. 12118719 10.1017/s0031182002001749

[38] Altschul SF, Gish W, Miller W, Myers EW, Lipman DJ. Basic local alignment search tool. J Mol Biol. 1990;215:403–10. 10.1016/S0022-2836(05)80360-2 2231712

[39] Lydersen S, Fagerland MW, Laake P. Recommended tests for association in 2 x 2 tables. Stat Med. 2009;28:1159–75. 10.1002/sim.3531 19170020

[40] Charrad M, Ghazzali N, Boiteau V, Niknafs A. NbClust: An R Package for Determining the Relevant Number of Clusters in a Data Set. J Stat Softw. 2014;61:36.

[41] Svobodova M, Vorisek P, Votypka J, Weidinger K. Heteroxenous coccidia (Apicomplexa: Sarcocystidae) in the populations of their final and intermediate hosts: European buzzard and small mammals. Acta Protozool. 2004;43:251–60.

[42] Fichet-Calvet E, Kia EB, Giraudoux P, Quere JP, Delattre P, Ashford RW. *Frenkelia* parasites in a small mammal community. Dynamics of infection and effect on the host. Parasite. 2004;11:301–10. 10.1051/parasite/2004113301 15490755

[43] Herrmann DC, Maksimov P, Maksimov A, Sutor A, Schwarz S, Jaschke W, et al *Toxoplasma gondii* in foxes and rodents from the German Federal States of Brandenburg and Saxony-Anhalt: seroprevalence and genotypes. Vet Parasitol. 2012;185:78–85. 10.1016/j.vetpar.2011.10.030 22105083

[44] Hurkova-Hofmannova L, Qablan MA, Jurankova J, Modry D, Pialek J. A survey of *Toxoplasma gondii* and *Neospora caninum* infecting house mice from a hybrid zone. J Parasitol. 2014;100(1):139–41. 10.1645/13-255.1 23927367

[45] Hughes JM, Williams RH, Morley EK, Cook DA, Terry RS, Murphy RG, et al The prevalence of *Neospora caninum* and co-infection with *Toxoplasma gondii* by PCR analysis in naturally occurring mammal populations. Parasitology. 2006;132:29–36. 10.1017/S0031182005008784 16393351

[46] Meerburg BG, De Craeye S, Dierick K, Kijlstra A. *Neospora caninum* and *Toxoplasma gondii* in brain tissue of feral rodents and insectivores caught on farms in the Netherlands. Vet Parasitol. 2012;184:317–20. 10.1016/j.vetpar.2011.09.001 21958437

[47] Reperant LA, Hegglin D, Tanner I, Fischer C, Deplazes P. Rodents as shared indicators for zoonotic parasites of carnivores in urban environments. Parasitology. 2009;136:329–37. 10.1017/S0031182008005428 19154652

[48] Fuehrer HP, Bloschl I, Siehs C, Hassl A. Detection of *Toxoplasma gondii*, *Neospora caninum*, and *Encephalitozoon cuniculi* in the brains of common voles (*Microtus arvalis*) and water voles (*Arvicola terrestris*) by gene amplification techniques in western Austria (Vorarlberg). Parasitol Res. 2010;107:469–73. 10.1007/s00436-010-1905-z 20480373

[49] Thomasson D, Wright EA, Hughes JM, Dodd NS, Cox AP, Boyce K, et al Prevalence and co-infection of *Toxoplasma gondii* and *Neospora caninum* in *Apodemus sylvaticus* in an area relatively free of cats. Parasitology. 2011;138:1117–23. 10.1017/S0031182011000904 21756421

[50] Staubach C, Thulke HH, Tackmann K, Hugh-Jones M, Conraths FJ. Geographic information system-aided analysis of factors associated with the spatial distribution of *Echinococcus multilocularis* infections of foxes. Am J Trop Med Hyg. 2001;65:943–8. 11792003 10.4269/ajtmh.2001.65.943

[51] Denzin N, Schliephake A, Fröhlich A, Ziller M, Conraths FJ. On the move? *Echinococcus multilocularis* in red foxes of Saxony-Anhalt (Germany). Transbound Emerg Dis. 2014;61:239–46. 10.1111/tbed.12026 23134586

[52] Staubach C, Hoffmann L, Schmid VJ, Ziller M, Tackmann K, Conraths FJ. Bayesian space-time analysis of *Echinococcus multilocularis*-infections in foxes. Vet Parasitol. 2011;179:77–83. 10.1016/j.vetpar.2011.01.065 21367526

[53] Führer HP, Schneider R, Walochnik J, Auer H. Extraintestinal helminths of the common vole (*Microtus arvalis*) and the water vole (*Arvicola terrestris*) in Western Austria (Vorarlberg). Parasitol Res. 2010;106:1001–4. 10.1007/s00436-010-1753-x 20148339

[54] Hrckova G, Miterpakova M, O'Connor A, Snabel V, Olson PD. Molecular and morphological circumscription of *Mesocestoides* tapeworms from red foxes (*Vulpes vulpes*) in central Europe. Parasitology. 2011;138:638–47. 10.1017/S0031182011000047 21349216

[55] Zalesny G, Hildebrand J. Molecular identification of *Mesocestoides* spp. from intermediate hosts (rodents) in central Europe (Poland). Parasitol Res. 2012;110:1055–61. 10.1007/s00436-011-2598-7 21847599

[56] Eberwein P, Haeupler A, Kuepper F, Wagner D, Kern WV, Muntau B, et al Human infection with marten tapeworm. Emerg Infect Dis. 2013;19:1152–4. 10.3201/eid1907.121114 23763821 PMC4816405

[57] Brunet J, Benoilid A, Kremer S, Dalvit C, Lefebvre N, Hansmann Y, et al First case of human cerebral *Taenia martis* cysticercosis. J Clin Microbiol. 2015;53:2756–9. 10.1128/JCM.01033-15 26019196 PMC4508436

[58] Brunet J, Pesson B, Chermette R, Regnard P, Grimm F, Deplazes P, et al First case of peritoneal cysticercosis in a non-human primate host (*Macaca tonkeana*) due to *Taenia martis*. Parasit Vectors. 2014;7:422 10.1186/1756-3305-7-422 25189669 PMC4167275

[59] De Liberato C, Berrilli F, Meoli R, Friedrich KG, Di Cerbo P, Cocumelli C, et al Fatal infection with *Taenia martis* metacestodes in a ring-tailed lemur (*Lemur catta*) living in an Italian zoological garden. Parasitol Int. 2014;63:695–7. 10.1016/j.parint.2014.05.008 24928170

[60] Strube C, Heuer L, Janecek E. *Toxocara* spp. infections in paratenic hosts. Vet Parasitol. 2013;193:375–89. 10.1016/j.vetpar.2012.12.033 23312872

[61] Kazacos KR, Jelicks LA, Tanowitz HB. *Baylisascaris* larva migrans. Handb Clin Neurol. 2013;114:251–62. 10.1016/B978-0-444-53490-3.00020-0 23829916

[62] Havasiova K, Dubinsky P, Stefancikova A. A seroepidemiological study of human *Toxocara* infection in the Slovak Republic. J Helminthol. 1993;67(4):291–6. 8132974 10.1017/s0022149x00013298

[63] Jin Y, Shen C, Huh S, Choi MH, Hong ST. Cross-reactivity of Toxocariasis with Crude Antigen of *Toxascaris leonina* Larvae by ELISA. J Korean Med Sci. 2015;30:549–51. 10.3346/jkms.2015.30.5.549 25931784 PMC4414637

[64] Zahabiun F, Sadjjadi SM, Yunus MH, Rahumatullah A, Moghaddam MH, Saidin S, et al Production of *Toxocara cati* TES-120 Recombinant Antigen and Comparison with its *T*. *canis* Homolog for Serodiagnosis of Toxocariasis. Am J Trop Med Hyg. 2015; 93:319–25. 10.4269/ajtmh.15-0190 26033026 PMC4530755

[65] Zibaei M, Sadjjadi SM, Sarkari B, Uga S. Evaluation of *Toxocara cati* Excretory-Secretory Larval Antigens in Serodiagnosis of Human Toxocariasis. J Clin Lab Anal. 2016; 30:248–53. 10.1002/jcla.21844 25846840 PMC6807093

[66] Rodriguez-Caballero A, Martinez-Gordillo MN, Medina-Flores Y, Medina-Escutia ME, Meza-Lucas A, Correa D, et al Successful capture of *Toxocara canis* larva antigens from human serum samples. Parasit Vectors. 2015;8:264 10.1186/s13071-015-0875-5 25952316 PMC4426178

[67] Antolova D, Reiterova K, Miterpakova M, Stanko M, Dubinsky P. Circulation of *Toxocara* spp. in suburban and rural ecosystems in the Slovak Republic. Vet Parasitol. 2004;126:317–24. 10.1016/j.vetpar.2004.08.005 15567594

[68] Morgan ER, Azam D, Pegler K. Quantifying sources of environmental contamination with *Toxocara* spp. eggs. Vet Parasitol. 2013;193:390–7. 10.1016/j.vetpar.2012.12.034 23333071

[69] Nijsse R, Mughini-Gras L, Wagenaar JA, Franssen F, Ploeger HW. Environmental contamination with *Toxocara* eggs: a quantitative approach to estimate the relative contributions of dogs, cats and foxes, and to assess the efficacy of advised interventions in dogs. Parasit Vectors. 2015;8:397 10.1186/s13071-015-1009-9 26216217 PMC4517363

