# Supplementary material for: Small rodents as paratenic or intermediate hosts of carnivore parasites in Berlin, Germany
Source: PLoS One. 2017 Mar 9;12(3):e0172829. doi: 10.1371/journal.pone.0172829 (PMC5344343; doi:10.1371/journal.pone.0172829)
Supplement: S1 Table — (DOCX) [file pone.0172829.s001.docx]

**S1 Table. Primer sequences and PCR conditions.**

| Parasites/ target | Name | Sequenz | Ta (°C)a | At (s)b | Et (s)c | Cycle no. | Size (bp) | References |
| --- | --- | --- | --- | --- | --- | --- | --- | --- |
| Ascaridae/ | F ITS 1 | GGCAAAAGTCGTAACAAGGT | 60 | 15 | 15 | 40 | nad | (Ishiwata et al., 2004) |
| ITS-1 | R ITS1 | CTGCAATTCGCACTATTTATCG |  |  |  |  |  |  |
| Toxoplasma | Tox 5 for | CGCTGCAGACACAGTGCATCTGGAT | 63 | 30 | 15 | 45 | 450 | (Homan et al., 2000; |
| gondii/  519 bp repeat | Tox 8 rev | CCCAGCTGCGTCTGTCGGGAT |  |  |  |  |  | Reischel et al., 2003) |
| Coccidia/ | Coccidian-ITS- | AAGTATAAGCTTTTATACGGCT | 62 | 30 | 15 | 40 | na | (Ho et al., 1996) |
| ITS-1 | 1-COC-1for |  |  |  |  |  |  |  |
|  | Coccidian-ITS- | CACTGCCACGGTAGTCCAATAAC |  |  |  |  |  |  |
|  | 1-COC-2rev |  |  |  |  |  |  |  |
| Plathelmithes/ | N2 | ATCGACATCTTGAACGCATATTGC | 62 | 15 | 30 | 40 | na | (Gasser & Chilton, 1995) |
| ITS-2 | NC6 | TTAGTTTCTTTTCCTCCGCT |  |  |  |  |  |  |
| Plathelminthes/ | Plathelm12Sfor | TTAAGATATATGTGGTACAGGATTAGATACCC | 63 | 15 | 15 | 40 | 314 | (von Nickisch-Rosenegk |
| 12S rRNA gene |  |  |  |  |  |  |  | et al., 1999 |
|  | Plathelm12Srev | AACCGAGGGTGACGGGCGGTGTGTACC |  |  |  |  |  |  |
| Plathelminthes/ | PlathelCOIfor | TTTTTTGGGCATCCTGAGGTTTAT | 61 | 15 | 15 | 40 | 417 | (Bowles et al., 1992) |
| COI gene |  |  |  |  |  |  |  |  |
|  | PlathelCOIrev | TAAAGAAAGAACATAATGAAAATG |  |  |  |  |  |  |

aAnnealing temperature

bAnnealing time

cExtention time

dnot available due to species-specific differences
