# Supplementary material for: Small rodents as paratenic or intermediate hosts of carnivore parasites in Berlin, Germany
Source: PLoS One. 2017 Mar 9;12(3):e0172829. doi: 10.1371/journal.pone.0172829 (PMC5344343; doi:10.1371/journal.pone.0172829)
Supplement: S2 Table — (DOCX) [file pone.0172829.s002.docx]

**S2 Table. Parasite prevalences in *Apodemus flavicollis.***

|  | *Frenkelia glareoli* PCR  Number  % Prevalence (95% CI^a^) | *Toxoplasma gondii* PCR  Number  % Prevalence (95% CI) | *Toxocara canis* PCR  Number  % Prevalence (95% CI) | *Toxocara canis* ELISA  Number  % Prevalence (95% CI) |
| --- | --- | --- | --- | --- |
| All | 82  8.5 (4.2-16.6) | 82  2.4 (0.7-8.5) | 82  3.7 (1.3-10.2) | 77  1.3 (0.2-7.0) |
| Juvenile | 0 | 0 | 0 | 0 |
| Subadult^b^ | 8  12.5 (2.2-47.1) | 8  0 (0-25.3) | 8  0 (0-25.3) | 7  0 (0-27.9) |
| Adult | 74  8.1 (3.8-16.6) | 74  2.7 (0.7-9.3) | 74  4.1 (1.4-11.3) | 70  1.4 (0.3-7.7) |
| Female | 41  12.2 (5.3-25.5) | 41  2.4 (0.4-12.6) | 41  4.9 (1.3-16.1) | 38  2.6 (0.5-13.5) |
| Male | 41  4.9 (1.3-16.1) | 41  2.4 (0.4-12.6) | 41  2.4 (0.4-12.6) | 39  0 (0-9.0) |
| Gatow | 33  15.2 (6.7-30.9) | 33  0 (0-10.4) | 33  3.0 (0.5-15.3) | 33  0 (0-10.4) |
| Tegel | 22  4.5 (0.8-21.8) | 22  9.1 (2.5-27.8) | 22  4.5 (0.8-21.8) | 23  4.8 (0.8-22.7) |
| Moabit | 0 | 0 | 0 | 0 |
| Steglitz | 27  3.7 (0.7-18.3) | 27  0 (0-12.5) | 27  3.7 (0.7-18.3) | 21  0 (0-14.3) |

^a^95% confidence interval

^b^Full-grown animals without signs of sexual activity
