# Supplementary material for: Small rodents as paratenic or intermediate hosts of carnivore parasites in Berlin, Germany
Source: PLoS One. 2017 Mar 9;12(3):e0172829. doi: 10.1371/journal.pone.0172829 (PMC5344343; doi:10.1371/journal.pone.0172829)
Supplement: S3 Table — (DOCX) [file pone.0172829.s003.docx]

**S3 Table. Parasite prevalences in *Apodemus sylvaticus.***

|  | *Frenkelia glareoli* PCR  Number  % Prevalence (95% CI^a^) | *Toxoplasma gondii* PCR  Number  % Prevalence (95% CI) | *Toxocara canis* PCR  Number  % Prevalence (95% CI) | *Toxocara canis* ELISA  Number  % Prevalence (95% CI) |
| --- | --- | --- | --- | --- |
| All | 25  0 (0-13.2) | 25  8.0 (2.2-25.0) | 25  8.0 (2.2-25.0) | 25  28.0 14.3-47.6) |
| Juvenile | 2  0 (0-65.8) | 2  0 (0-65.8) | 2  50 (2.5-97.5) | 2  0 (0-65.8) |
| Subadult^b^ | 2  0 (0-65.8) | 2  0 (0-65.8) | 2  0 (0-65.8) | 2  50 (2.5-97.5) |
| Adult | 21  0 (0-15.5) | 21  9.5 (2.7-28.9) | 21  4.8 (0.8-22.7) | 21  28.6 (13.8-50.0) |
| Female | 11  0 (0-25-9) | 11  0 (0-25-9) | 11  9.1 (1.6-37.7) | 11  36.4 (15.2-64.6) |
| Male | 14  0 (0-21.5) | 14  14.3 (4.0-39.9) | 14  7.1 (1.3-31.5) | 14  21.4 (7.6-47.6) |
| Gatow | 0 | 0 | 0 | 0 |
| Tegel | 0 | 0 | 0 | 0 |
| Moabit | 25  0 (0-13.2) | 25  8.0 (2.2-25.0) | 25  8.0 (2.2-25.0) | 25  28.0 14.3-47.6) |
| Steglitz | 0 | 0 | 0 | 0 |

^a^95% confidence interval

^b^Full-grown animals without signs of sexual activity
