# Supplementary material for: Small rodents as paratenic or intermediate hosts of carnivore parasites in Berlin, Germany
Source: PLoS One. 2017 Mar 9;12(3):e0172829. doi: 10.1371/journal.pone.0172829 (PMC5344343; doi:10.1371/journal.pone.0172829)
Supplement: S4 Table — (DOCX) [file pone.0172829.s004.docx]

**S4 Table. Parasite prevalences in *Apodemus agrarius.***

|  | *Frenkelia glareoli* PCR  Number  % Prevalence (95% CI^a^) | *Toxoplasma gondii* PCR  Number  % Prevalence (95% CI) | *Toxocara canis* PCR  Number  % Prevalence (95% CI) | *Toxocara canis* ELISA  Number  % Prevalence (95% CI) |
| --- | --- | --- | --- | --- |
| All | 78  1.3 (0.2-6.9) | 78  5.1 (2.0-12.5) | 78  3.8 (1.3-10.7) | 72  30.6 (21.1-42.0) |
| Juvenile | 4  0 (0-49.0) | 4  0 (0-49.0) | 4  0 (0-49.0) | 3  0 (0-56.2) |
| Subadult^b^ | 9  0 (0-29.9) | 9  0 (0-29.9) | 9  0 (0-29.9) | 8  25 (7.1-59.1) |
| Adult | 65  0 (0-5.6) | 65  6.2 (2.4-14.8) | 65  4.6 (1.6-12.7) | 61  32.8 (22.3-45.3) |
| Female | 34  2.9 (0.5-14.9) | 34  5.9 (1.6-19.1) | 34  2.9 (0.5-14.9) | 31  38.7-23.7-56.1) |
| Male | 44  0 (0-8.0) | 44  4.5 (1.3-15.1) | 44  4.5 (1.3-15.1) | 41  24.4 (13.8-39.3) |
| Gatow | 5  0 (0-43.5) | 5  20 (3.6-62.4) | 5  0 (0-43.5) | 5  60 (23-1-88.2) |
| Tegel | 0 | 0 | 0 | 0 |
| Moabit | 0 | 0 | 0 | 0 |
| Steglitz | 73  1.4 (0.2-7.4) | 73  4.1 (1.4-11.4) | 73  6.8 (3.0-15.1) | 67  28.4 (19.0-40.1) |

^a^95% confidence interval

^b^Full-grown animals without signs of sexual activity
