# Supplementary material for: Small rodents as paratenic or intermediate hosts of carnivore parasites in Berlin, Germany
Source: PLoS One. 2017 Mar 9;12(3):e0172829. doi: 10.1371/journal.pone.0172829 (PMC5344343; doi:10.1371/journal.pone.0172829)
Supplement: S5 Table — (DOCX) [file pone.0172829.s005.docx]

**S5 Table. Parasite prevalences in *Myodes glareolus.***

|  | *Frenkelia glareoli* PCR  Number  % Prevalence (95% CI^a^) | *Toxoplasma gondii* PCR  Number  % Prevalence (95% CI) | *Toxocara canis* PCR  Number  % Prevalence (95% CI) | *Toxocara canis* ELISA  Number  % Prevalence (95% CI) |
| --- | --- | --- | --- | --- |
| All | 60  43.3 (31.6-55.9) | 60  0 (0-4.3) | 60  5.0 (1.7-13.7) | 56  3.6 (1.0-12.1) |
| Juvenile | 1  100 (27.0-100) | 1  0 (0-73.0) | 1  100 (27.0-100) | 1  100 (27.0-100) |
| Subadult^b^ | 2  0 (0-65.8) | 2  0 (0-65.8) | 2  0 (0-65.8) | 2  0 (0-65.8) |
| Adult | 55  43.6 (31.4-56.7) | 55  0 (0-6.5) | 55  3.6 (1.0-12.3) | 52  1.9 (0.3-10.1) |
| Female | 29  41.4 (25.5-59.3) | 29  0 (0-11.7) | 29  3.4 (0.6-17.2) | 27  3.7 (0.7-18.3) |
| Male | 30  46.7 (30.2-63.9) | 30  0 (0-11.4) | 30  6.7 (11.8-21.3) | 29  3.4 (0.6-17.2) |
| Gatow | 45  38.6 (25.7-53.4) | 45  0 (0-7.9) | 45  0 (0-7.9) | 44  0 (0-8.0) |
| Tegel | 15  60 (35.8-80.2) | 15  0 (0-20.4) | 15  20 (7.0-45.2) | 13  15.4 (4.3-42.2) |
| Moabit | 0 | 0 | 0 | 0 |
| Steglitz | 0 | 0 | 0 | 0 |

^a^95% confidence interval

^b^Full-grown animals without signs of sexual activity
