# Supplementary material for: Small rodents as paratenic or intermediate hosts of carnivore parasites in Berlin, Germany
Source: PLoS One. 2017 Mar 9;12(3):e0172829. doi: 10.1371/journal.pone.0172829 (PMC5344343; doi:10.1371/journal.pone.0172829)
Supplement: S6 Table — (DOCX) [file pone.0172829.s006.docx]

**S6 Table. Parasite prevalences in *Microtus arvalis.***

|  | *Frenkelia glareoli* PCR  Number  % Prevalence (95% CI^a^) | *Toxoplasma gondii* PCR  Number  % Prevalence (95% CI) | *Toxocara canis* PCR  Number  % Prevalence (95% CI) | *Toxocara canis* ELISA  Number  % Prevalence (95% CI) |
| --- | --- | --- | --- | --- |
| All | 11  0 (0-25.9) | 11  27.3 (9.7-56.6) | 11  0 (0-25.9) | 9  0 (0-37.1) |
| Juvenile | 0 | 0 | 0 | 0 |
| Subadult^b^ | 0 | 0 | 0 | 0 |
| Adult | 11  0 (0-25.9)0 | 11  27.3 (9.7-56.6) | 11  0 (0-25.9) | 11  0 (0-25.9)0 |
| Female | 0 | 7  42.9 (12.3-78.4) | 0 | 0 |
| Male | 0 | 4  0 (0-60.4) | 0 | 0 |
| Gatow | 11  0 (0-25.9) | 11  27.3 (9.7-56.6) | 11  0 (0-25.9) | 9  0 (0-37.1) |
| Tegel | 0 | 0 | 0 | 0 |
| Moabit | 0 | 0 | 0 | 0 |
| Steglitz | 0 | 0 | 0 | 0 |

^a^95% confidence interval

^b^Full-grown animals without signs of sexual activity
