# Supplementary material for: Small rodents as paratenic or intermediate hosts of carnivore parasites in Berlin, Germany
Source: PLoS One. 2017 Mar 9;12(3):e0172829. doi: 10.1371/journal.pone.0172829 (PMC5344343; doi:10.1371/journal.pone.0172829)
Supplement: S7 Table — (DOCX) [file pone.0172829.s007.docx]

**S7 Table. Parasite prevalences in *Microtus agrestis.***

|  | *Frenkelia glareoli* PCR  Number  % Prevalence (95% CI^a^) | *Toxoplasma gondii* PCR  Number  % Prevalence (95% CI) | *Toxocara canis* PCR  Number  % Prevalence (95% CI) | *Toxocara canis* ELISA  Number  % Prevalence (95% CI) |
| --- | --- | --- | --- | --- |
| all | 2  0 (0-65.8) | 2  50 (2.5-97.5) | 2  0 (0-65.8) | 2  0 (0-65.8) |
| juvenile | 0 | 0 | 0 | 0 |
| subadult^b^ | 0 | 0 | 0 | 0 |
| adult | 0 | 2  50 (2.5-97.5) | 0 | 0 |
| female | 0 | 2  50 (2.5-97.5) | 0 | 0 |
| male | 0 | 0 | 0 | 0 |
| Gatow | 0 | 2  50 (2.5-97.5) | 0 | 0 |
| Tegel | 0 | 0 | 0 | 0 |
| Moabit | 0 | 0 | 0 | 0 |
| Steglitz | 0 | 0 | 0 | 0 |

^a^95% confidence interval

^b^Full-grown animals without signs of sexual activity
