# Supplementary material for: Small rodents as paratenic or intermediate hosts of carnivore parasites in Berlin, Germany
Source: PLoS One. 2017 Mar 9;12(3):e0172829. doi: 10.1371/journal.pone.0172829 (PMC5344343; doi:10.1371/journal.pone.0172829)
Supplement: S1 Information — (DOCX) [file pone.0172829.s011.docx]

**S1 Information: Detailed description of models for logistic regression of seroprevalence data for *Toxocara canis*.**

Separately, the variables species and location, significantly increased the odds to be positive for *T. canis* and *A. sylvaticus* and *A. agrarius* had about 3.5fold higher odds (p < 0.001 and p < 0.01, respectively) than *A. flavicollis* and with Moabit and Steglitz having odds ratios of 10.4 and 7.3 (p<0.01) compared to Gatow (Figure 2A).

A model including both explanatory variables, species and location, decreased the AIC from 178.74 (only location) and 158.21 (only rodent species) to 153.15, the lowest value for all models considered for *T. canis* seropositivity. This model also had by far the best pseudo-R^2^ value of 0.28 and a LRT versus the models containing only one of the parameters showed a significant improvement due to the combination of variables. However, in this combination, 95% CI for odds ratios were extremely wide and no variable level on its own had a significant effect on the probability to be positive in the *T. canis* ES antigen ELISA. Furthermore, none of the variables had a significant influence on probability to detect antibodies in a Wald test (p = 0.58 for study location and p = 0.95 for species). Therefore, the genus was considered instead of species.

Inclusion of the variable genus as only variable in the model resulted in an AIC of 183.23 and *Myodes* had a significantly lower odds (decreased by approximately 5.6 fold) than *Apodemus* (p = 0.02) (Fig. 2B). Adding the variable study site improved the AIC (180.22) but in this model only the locations Moabit and Steglitz had a significant influence on the odds (Fig. 2C) but the genera were not associated with a significant p value suggesting that the study location is more important than the rodent genus although both variables are strongly co-linear. However, Wald tests conducted to identify variables with a significance influence on the odds to be seropositive for *T. canis* revealed no significant influences of location or genus on the probability to detect *T. canis* antibodies (i.e. 0.75 for genus and 0.074 for study site). The pseudo-R^2^ for this model is also quite poor (0.11) and a LRT comparing these two models revealed that the model with the parameters genus and study location was not significantly better than the model containing only the parameter location (p = 0.28). However, the model with both variables was significantly better than the model considering only the variable genus (p = 0.011) in the LRT.

If only the variable location was included, influence points varied between 2.7 and 7.1 for the positive animals and were below 0.65 for the negative ones. In the model with genus as the only variable included, influence points for positive animals were 7.2 (two animals) or 3.1 whereas negative animals had influence points below 0.4. In the model considering both variables, influence points between 7.1 (one animal) and 2.7 were observed for *T. canis* positive rodents. Two of the negative animals had influence points of 2.2 while the rest was lower than 0.7.
